# Cold-Setting Inkjet Printed Titania Patterns Reinforced by Organosilicate Binder

**DOI:** 10.3390/molecules200916582

**Published:** 2015-09-11

**Authors:** Marcela Králová, Petr Dzik, Vít Kašpárek, Michal Veselý, Jaroslav Cihlář

**Affiliations:** 1 Central European Institute of Technology, Brno University of Technology, Technicka 3058/10, Brno 61600, Czech Republic; E-Mails: marcela.kralova@ceitec.vutbr.cz (M.K.); vit.kasparek@ceitec.vutbr.cz (V.K.); jaroslav.cihlar@ceitec.vutbr.cz (J.C.); 2 Faculty of Chemistry, Brno University of Technology, Purkynova 118, Brno 61200, Czech Republic; E-Mail: vesely-m@fch.vutbr.cz

**Keywords:** titania, silica binder, material printing, direct patterning, UV-curing

## Abstract

A hybrid organo-silica sol was used as a binder for reinforcing of commercial titanium dioxide nanoparticles (Evonic P25) deposited on glass substrates. The organo-silica binder was prepared by the sol-gel process and mixtures of titania nanoparticles with the binder in various ratios were deposited by materials printing technique. Patterns with both positive and negative features down to 100 µm size and variable thickness were reliably printed by Fujifilm Dimatix inkjet printer. All prepared films well adhered onto substrates, however further post-printing treatment proved to be necessary in order to improve their reactivity. The influence of UV radiation as well as of thermal sintering on the final electrochemical and photocatalytic properties was investigated. A mixture containing 63 wt % of titania delivered a balanced compromise of mechanical stability, generated photocurrent density and photocatalytic activity. Although the heat treated samples yielded generally higher photocurrent, higher photocatalytic activity towards model aqueous pollutant was observed in the case of UV cured samples because of their superhydrophilic properties. While heat sintering remains the superior processing method for inorganic substrates, UV-curing provides a sound treatment option for heat sensitive ones.

## 1. Introduction

Environmental pollution is becoming very serious problem and the demand for safe drinking water is continuously increasing with increasing population growth. Disinfection and decontamination are considered as conventional methods for water treatment. Their main disadvantages are energetic and operational demands, requirement of intensive chemical treatments the residuals of which can even increase the problems with pollution and decontamination [1]. New techniques such as advanced oxidation processes could improve the effectivity of these traditional water treatment processes because of their lower energy demands and less chemicals used [2]. Photocatalysis is one such method applicable not only for water treatment, but also for air cleaning and self-cleaning solid surfaces. Its principle is based on electrons and holes separation caused by the absorption of UV light and their diffusion to the semiconductors surface where they can take part in various red-ox reactions. During the past years, this technique has been widely applied in environmental purification tasks [3,4] as well as in artificial photosynthesis [5]. Titanium dioxide has become the most investigated and the most prominently used photocatalyst [6,7] because of its relatively high photoactivity, biological and chemical inertness, competitive price and creation of stable solutions [8].

Generally, the employment of freely suspended powder photocatalysts brings some disadvantages such as strong tendency to aggregation, low adsorption capacity, sedimentation and deposit formation [9]. Moreover, the unavoidable need of catalyst separation from the solution after the treatment and can prohibitively complicate large scale processes. To overcome these drawbacks, the photocatalyst can be immobilized by the anchoring onto various substrates. Titania coatings have been successfully fabricated by both gas-phase methods [10,11,12] as well as wet-coating methods [13,14,15] and both approaches resulted into highly active immobilized photocatalysts [16].

Recently, the traditional wet-coating methods such as spin-coating, dip-coating, doctor blade coating *etc*., have been challenged by a new approach most often termed as material printing [17]. The process essentially involves the deposition of layers and patterns by means of traditional printing techniques where conventional color inks have been replaced by special functional inks. Strictly speaking, this technique has been around for the past 60 years but was actually limited to screen printing of pulverized metal pigment inks during the fabrication of conductive tracks in the electronic industry [18]. During the past decade, the concept of material printing has been intensively developed and the portfolio of applicable printing techniques has broadened significantly [19].

While essentially all traditional printing techniques can be adopted for printing of functional patterns, inkjet printing [20] occupies quite a prominent position. Despite quite narrow viscosity and particle size limits, it is most suitable for lab-scale prototype development or customized production as no hardware printing form is required, *i.e*., patterns designed as computer files can be printed directly. Moreover, up-scaling from the lab level is easily accomplished merely by switching to a bigger format printer. Inkjet printing has been successfully used for the deposition of a variety of functional liquids, such as conducting polymers [21,22,23], dispersions of catalyst nanoparticles [24], *etc*. Numerous biochemical applications have been reported, such as tissue scaffolds [25,26] or cellular patterns [27,28]. Moreover, many prototype multicomponent devices such as capacitors [29], transistors [30], electronic filters [31], and organic light-emitting diodes [32] have been successfully fabricated. Recently we reported on a fully printed semiconductor-based photoelectrochemical cell [33].

Titania-silica mixtures represent a perspective group of materials that have recently been used as catalysts and supporting materials [34,35]. Titania-silica mixtures were found to improve the photocatalytic properties of pure titanium dioxide [36], increase the specific surface area [37] and improve the adsorption properties [38]. However, in order to be able to deposit the titania-silica film by material printing, an ink with appropriate properties needs to be formulated. Two distinct approaches to this task can be easily identified: 

The first one is based on the sol-gel process, *i.e.*, soluble titanium salts and/or titanium alkoxides are complexed by suitable chelates, pre-crosslinked by partial hydrolysis and the resulting metastable colloidal sols are then printed onto a substrate, gelled and converted into dense or porous oxide layer [39].

The second approach is based on the synthesis of stable colloidal suspension of nanocrystalline TiO_2_ followed by the delivery of this suspension onto a substrate by some of the numerous material printing techniques. Bernacka-Wojcik and coworkers recently demonstrated [40] a disposable biosensor integrating an inkjet printed photodetector fabricated by printing a commercial dispersion of titania particles with a desk-top office printer equipped with a thermal inkjet head. A similar approach was adopted by Yang *et al*. [41] who used a dispersion of TiO_2_ printed by a modified office inkjet printer to produce an oxygen demand sensing photoanode and by Arin *et al*. [42] who fabricated photocatalytically active TiO_2_ films by inkjet printing of nanoparticle suspensions obtained from microwave-assisted hydrothermal synthesis. Inkjet printing has been successfully employed also for the deposition of doped titania nanoparticles [43] as well as for the fabrication of DSSCs [44] titania photoanodes. Various methods have been utilized for the dispersing and stabilization of ultrafine printable suspensions, including ultrasound [45], microemulsions [46] and co-solvent mixtures [47]. The authors of this paper have recently reported the fabrication of titania patterns by inkjet printing of rutile nanodispersions originating from hydrothermal processes [48] and also the fabrication of printed composite titania-silica photoanodes [49].

The key difference between these two discussed approaches is determined by the post-deposition treatment requirements. The sol-gel originated coatings generally require oxidative heat processing in order to remove the organic fraction of the sol and induce crystallization of the oxide being created, while the suspension originated layers do not require heat treatment *per se* since prefabricated crystalline particles had been deposited. Yet, some form of further treatment and/or the presence of a binder may be necessary even in the case of the suspension originated coatings in order to ensure sufficient mechanical stability, adhesion to substrate and/or processing of further components present in the coating. However, if the treatment does not involve excessive heating, the deposition onto organic polymeric substrates becomes possible.

In this paper, we report on the fabrication of titanium dioxide layers by direct inkjet pattering of nanocrystalline suspension. Recently reported organo-silica binder [50] was mixed with commercial titania nanoparticles in order to improve printed layer mechanical properties. Inkjet printable formulations with various ratios of titania to binder were formulated and patterns of different thickness were printed onto soda-lime, Pyrex and FTO glass substrates. Printed green body patterns were further processed by thermal treatment or UV curing. The physical, chemical, electrical and photocatalytic properties of printed, sintered and UV-cured patterns were in detail investigated and the influence of post-deposition processing on printed layer properties was elucidated.

## 2. Results and Discussion

### 2.1. Printed Layers Imaging

**Figure 1:**
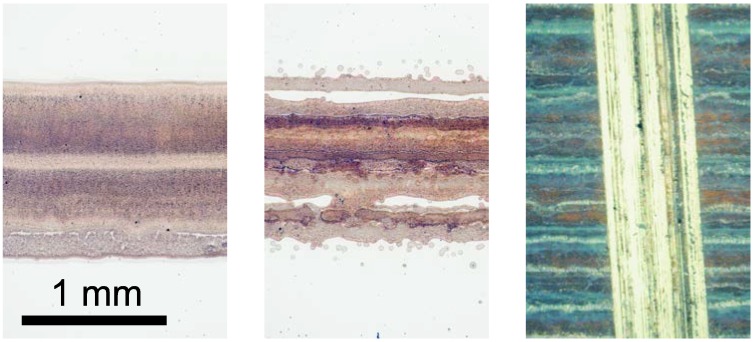
Optical micrographs of the printed layers showing the influence of ink composition on print quality. Formulation Ti_1 (**left**) featured good layer uniformity and excellent edge definition, but formulation Ti_5 (**middle**) suffered from occasional nozzle blockage and diverted droplets. The **right** image depicts a 3-layered patch of Ti_3 formulation featuring the banding artifacts and a scratch made with the pencil just harder than the layers itself.

For quick on-the-fly checking as well as the whole printed area inspection, optical microscopy and photomicrography are the preferred tools. First, one-layered samples of all tested formulations were compared in order to evaluate the printability of used formulations and substrate interactions. We observed that although all tested formulations were jettable, with increasing titania fraction the print quality worsened. Apparently, the silica binder helps to stabilize the titania particles, preventing aggregation and also improving the flow of the suspension during hydrodynamic stress accompanying transport through the print head. While the print quality of Ti_1 and Ti_2 formulations was essentially flawless, occasional nozzle blockage and/or diverted droplets occurred more often in the case of Ti_3 and Ti_4. Ti_5 required regular nozzle purging and cleaning to ensure acceptable print quality (Figure 1).

Multilayer printing was performed in the wet-to-dry manner, *i.e*., the previous layer was completely dry before printing the following one started. No problems such as bleeding or mottling which are usually associated with this procedure were observed here and the inks proved to be well suited for fabrication of thick patterns by multiple overprinting. However, after comparing the full range of studied layer thicknesses, we found out that with increasing number of layers banding artifacts started to develop at the boundaries of individual bands following the scanning motion of the printhead. The phenomenon was further studied by profilometry and resolved by adjustment of the printing formulation composition (see below).

### 2.2. Pencil-Hardness Test

The hardness of thin layers was determined in the whole range of hardness according the standard. The hardness of pencil that was not able to scratch the film was considered as the hardness of the film. Figure 1 depicts the results of a 3-layered patch of Ti_3 formulation scratching with the pencil just harder than the layers itself. Printed layers as well as heat treated layers were investigated by this method and significant differences were found. We observed that increasing of silica content in the printing formulation (from Ti_5 to Ti_1) led to the formation of harder films in the case of both printed as well as sintered samples. Moreover, the films showed a higher hardness after sintering process that is probably caused by the creation of silica network during the thermal treatment process. The results are summarized in Table 1.

**Table 1:** Hardness data for 3-layered samples.

Sample Name	ISO 15184 Hardness	
	UV Cured	Sintered
Ti_1	6B	10H
Ti_2	8B	10H
Ti_3	8B	10H
Ti_4	8B	F
Ti_5	8B	5B

### 2.3. Thermogravimetric Analysis

**Figure 2:**
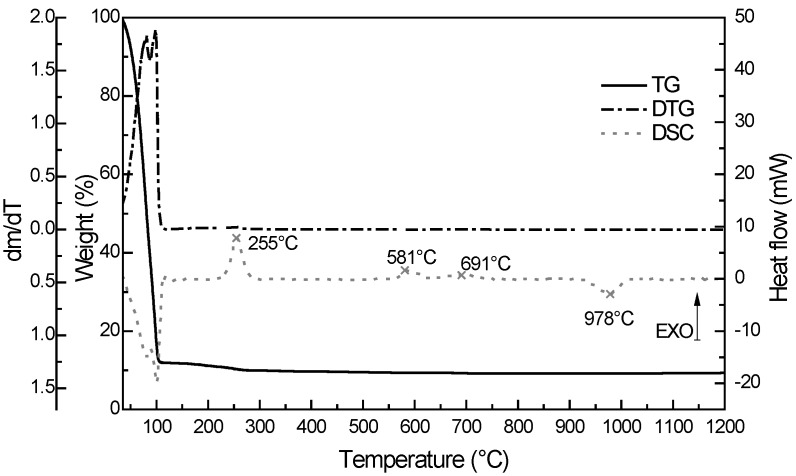
The record from thermogravimetric analysis for Ti_3.

Thermogravimetric behaviour was studied for all prepared printed mixtures. The samples shared common trends and a typical record is depicted at Figure 2. In the first temperature region (up to 120 °C), there was the highest weight loss which was attributed to the evaporation of adsorbed solvents (ethanol and butanol). This evaporation is represented by endothermic peak. The weight loss in the second temperature region (up to 315 °C) is related to the oxidation of CH_3_- groups from the MTEOS precursor. The oxidation is represented by exothermic peak at temperature around 255 °C. Any further weight loss cannot be observed beyond 315 °C. The other significant exothermic peak appeared at the temperature of 581 °C. This peak is associated with the phase transformation of amorphous SiO_2_ to tridymite [51]. Okada *et al*., discovered that silica matrix shift the temperature transformation of TiO_2_ from amorphous phase to anatase phase [52] so we supposed that in our case the phase transformation of anatase to rutile occurred also at higher temperature. Therefore, the exothermic peak at 691 °C is attributed to the mentioned transformation. The last peak at temperature 978 °C had endothermic character and it is connected with melting point of titania-silica system.

### 2.4. Profilometry

**Figure 3:**
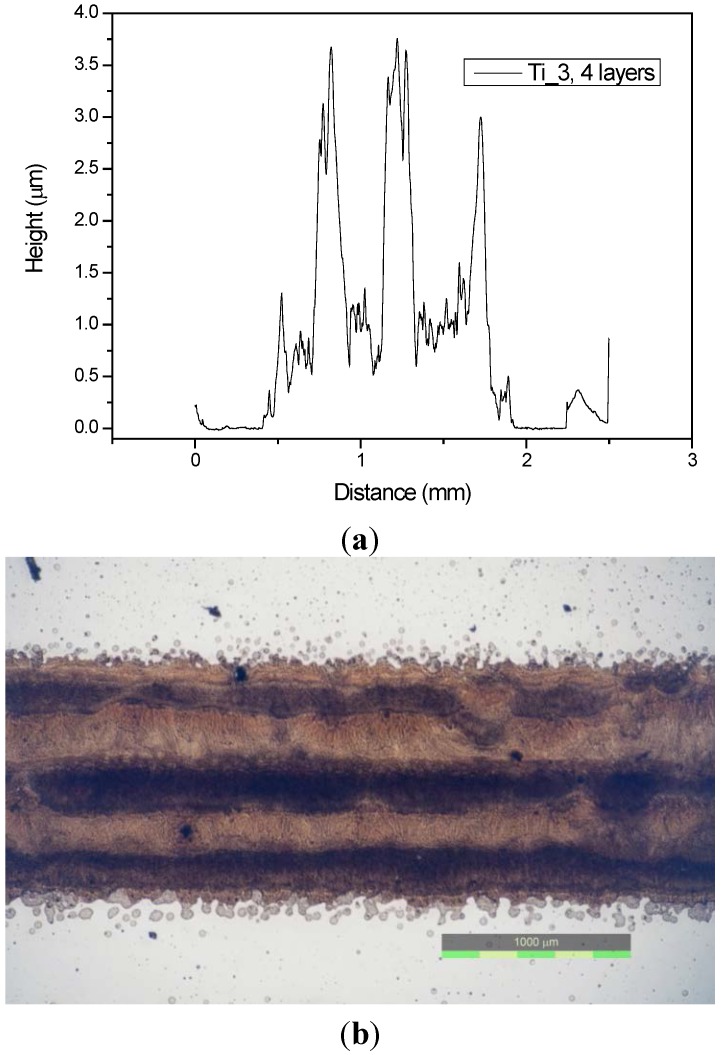
Banding patterns recorded in profilometric scan (**a**) and as seen in optical microscope (**b**).

During a preliminary testing, we discovered that there was virtually no influence of the processing mode on the thickness of printed patterns. All “green body” printed, sintered as well as UV-cured samples exhibited essentially identical values. This behavior was expected, since most of the printing formulation dry mass is constituted by titania nanoparticles forming a rigid structure permeated by the binder. Therefore, the profilometric thickness measurement was systematically conducted on the printed samples only (Figure 3).

Complete results of profilometric measurements for all samples are summarized in Table 2. The thickness is generally increasing with increasing titania content, with the exception of Ti_5 sample. We assume that the amount of printed ink in this case was actually lowered by occasional nozzle blockage. The RMS roughness calculated from a 3 mm scan length is relatively high with respect to the layer thickness. At such great scan length, it does not reflect the particle size but rather the banding artifacts observed in optical microscope and discussed above. Their geometrical distribution along the scan direction corresponds to the width of the printhead which confirms their cause and origin.

**Table 2:** Thickness and roughness of printed samples.

	Ti_1		Ti_2		Ti_3		Ti_4		Ti_5	
	Thickness (μm)	Rms (μm)	Thickness (μm)	Rms (μm)	Thickness (μm)	Rms (μm)	Thickness (μm)	Rms (μm)	Thickness (μm)	Rms (μm)
1L	0.512	0.252	0.555	0.239	0.573	0.263	0.766	0.480	0.748	0.282
2L	0.708	0.493	0.776	0.468	0.916	0.333	1.173	0.579	0.995	0.489
3L	1.024	0.771	1.214	0.700	1.330	0.741	1.708	1.017	1.273	0.727
4L	1.301	1.201	1.353	0.723	1.489	1.106	1.837	1.073	1.524	0.867
5L	1.746	1.588	1.763	1.092	1.906	1.113	2.224	1.275	2.008	1.089

### 2.5. Atomic Force Microscopy

**Figure 4:**
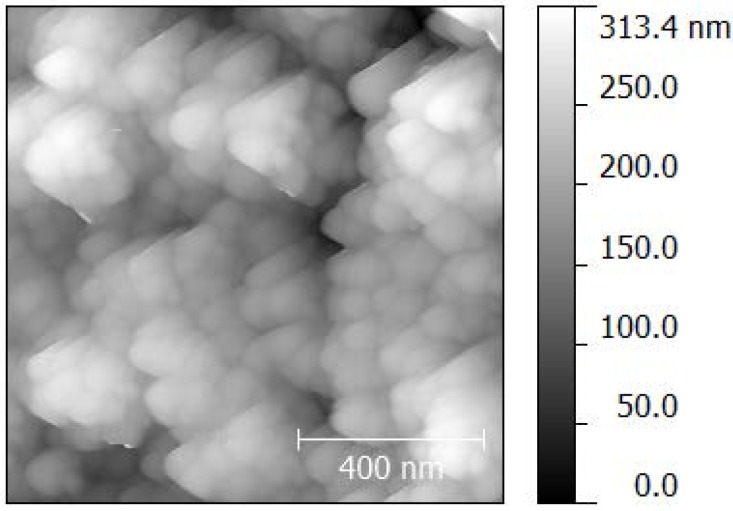
Topography of the sample Ti_4_P_4L.

The topography and roughness on the nanometer scale were analyzed by atomic force microscopy (Figure 4). Selected results for the three layered samples are summarized and compared with profilometric roughness in Table 3. On contrary to the profilometric measurements, the data obtained from AFM indicate much lower roughness of *ca*. 40 nm. With respect to the scan size (500 × 500 nm), we conclude that this figure includes the contribution of primary particles size (primary crystallite size of 21 nm is declared by the supplier) and the sub-milimeter artifacts which influenced the profilomeric roughness determination remain neglected by AFM. Moreover, we discovered that the roughness of all sample sets was decreasing with increasing layers count. Such behavior can be explained by the interaction of freshly printed material with the previous dry porous layer, filling the cavities created during drying of the previous one so the surface is more compact and the roughness is lower.

**Table 3:** RMS roughness (nm) of printed samples obtained from AFM.

Sample	Number of Layers				
	1L	2L	3L	4L	5L
Ti_1	60	49	39	28	26
Ti_2	74	45	39	40	34
Ti_3	76	45	43	42	40
Ti_4	78	55	53	40	37
Ti_5	74	38	28	27	21

### 2.6. Scanning Electron Microscopy

**Figure 5:**
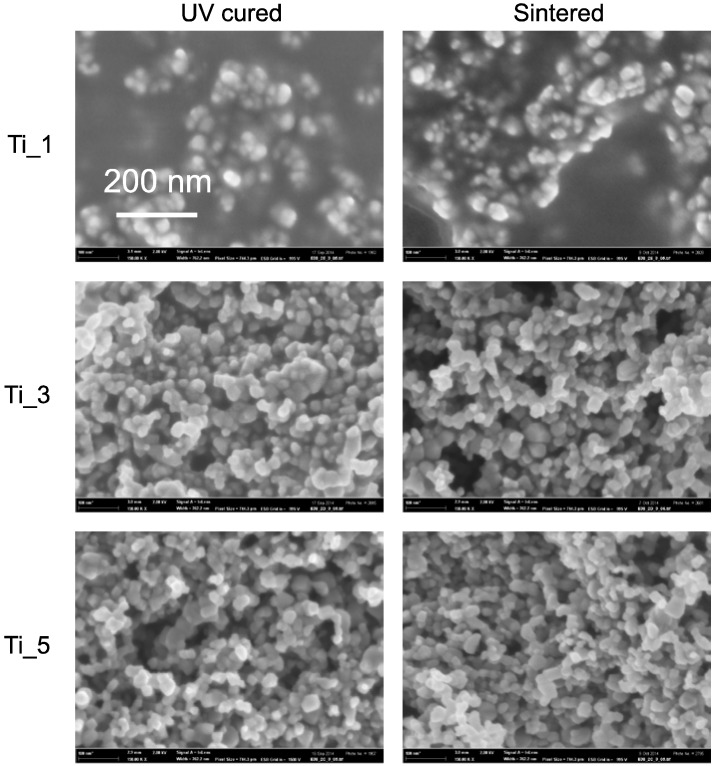
Images from SEM analysis; comparison of UV cured and sintered layers (3 layered samples).

The structure of the samples was examined by scanning electron microscopy. We discovered that there is no significant visual difference between the UV cured and sintered layers (Figure 5). The particle size in both cases was estimated between 20 and 40 nm which corresponds to the particle size declared for titania P25. It indicates that during the temperature treatment process, the particles were not subject to further aggregation. Moreover, we found that in case of Ti_1, where the fraction of TiO_2_ (Table 4) was the lowest, a discontinuous phase of titania particles forming isolated islands was formed (Figure 5, top). The other compositions with higher titania fraction seem to feature a rigid structure of communicating titania particles permeated by the binder (Figure 5, middle and bottom).

**Figure 6:**
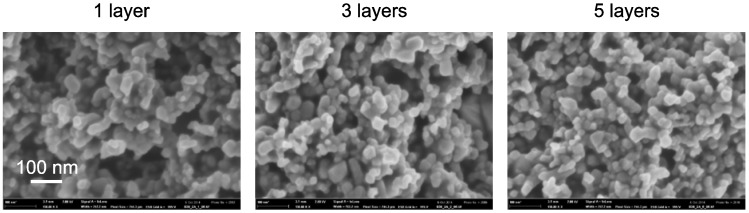
Flattening surface topology of sintered Ti_3 sample set with increasing number of printed layers as seen by SEM.

SEM images also allowed the comparison of different number layers Figure 6. We could observe the presence of pores both in the UV cured and thermally sintered samples and their frequency of occurrence was decreasing with increasing number of layers. This result was expected on the basis of previous AFM roughness determination. The recorded SEM images fully confirmed the suggested explanation of this phenomenon—decreasing roughness with increasing number of printed layers is caused by filling the cavities created during drying of the previous layer with freshly printed material.

### 2.7. Contact Angle Measurement

**Figure 7:**
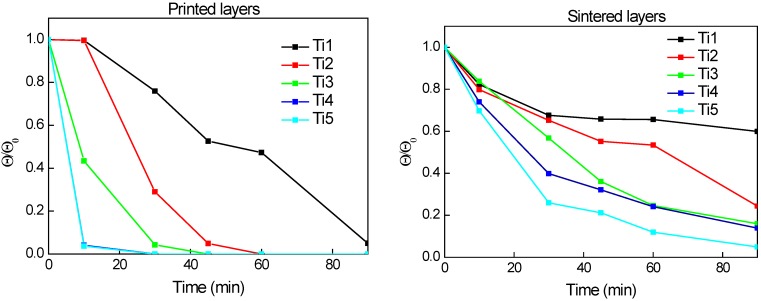

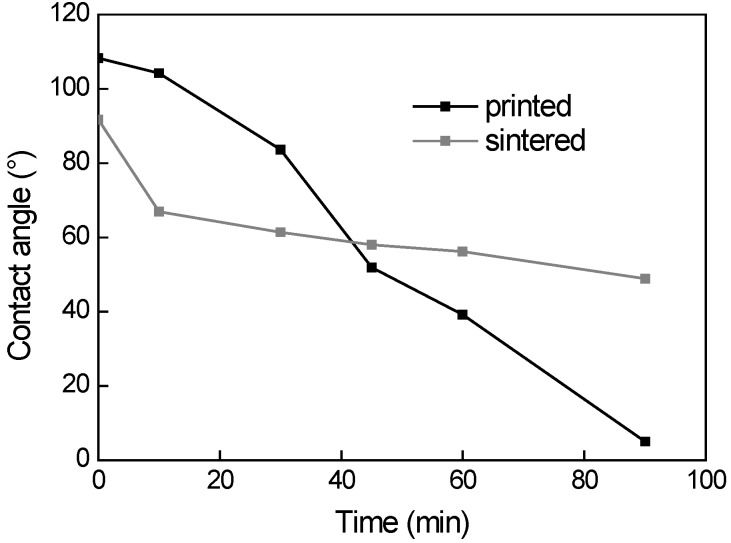
The dependence of relative contact angle on irradiation time for three layered samples. Printed “green body” layers (**top left**), thermally sintered layers (**top right**) and the comparison of absolute contact angle values for printed and sintered sample Ti_1 (**bottom**).

The photoinduced superhydrophility was examined by the sessile drop contact angle measurement on three layered samples. These samples were chosen according the results from optical and scanning electron microscopy. This study involved the comparison of printed and sintered sample sets. UV-cured sets were omitted as they exhibited superhydrophilic nature at the end of the curing process. We confirmed that printed layers had hydrophobic character which was caused by presence of methyl groups in silica binder [50]. Although these groups were inevitably oxidized during the sintering process, yet the character of these layers still remained hydrophobic. On the other hand, photocatalytic degradation of methyl groups which occurred during the post-printing UV curing caused changing the wetting properties to superhydrophilic. As superhydrophilic surface is considered that one where the water droplet applied on surface is immediately spread and its contact angle is lower than 5°. We discovered that the superhydrophilic wetting was reached after 90 min only for printed layers whereas this time was insufficient for sintered layers. When we compared all samples we found out that the contact angle decreased in an order depending on the amount of titania in the printing composition, with the greatest titania fraction sample being the fastest (Figure 7). This trend is observed for printed as well as for sintered layers.

### 2.8. Electrochemical Characterization

Linear sweep voltammetry has been used for the characterization of electrochemical properties. The photoelectrochemical response curve of the cell, *i.e*., the photocurrent generated plotted as a function of applied voltage, provides valuable information about the irradiated semiconductor properties. The depicted voltammograms represent the polarization curves of the studied sample in dark and under UV irradiation. In the positive potential range, the contribution of photogenerated current is obvious indicating free charge carrier formation and collection resulting from the absorption of UV quanta.

**Figure 8:**
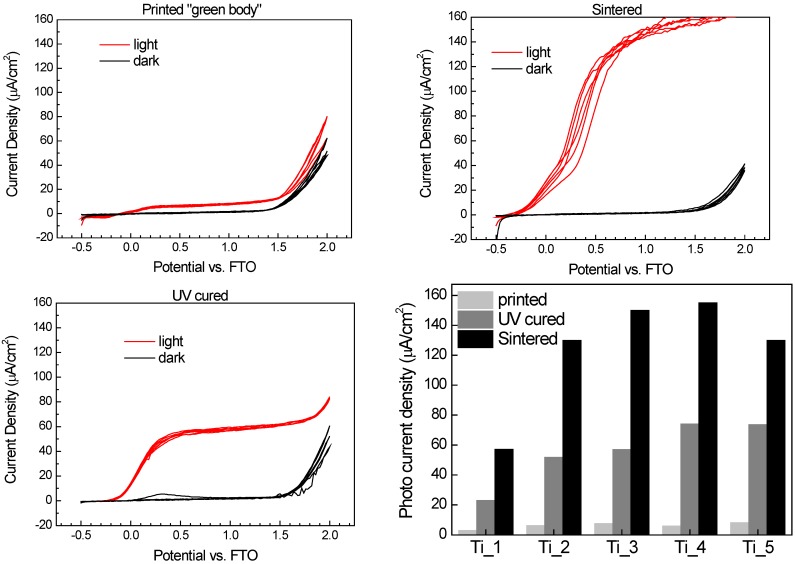
Comparison of generated photocurrents for Ti_3_3L sample. Printed “green body” electrode (**top left**), thermally sintered (**top right**), UV cured (**bottom left**) and the summary of photocurrent density (μA/cm^2^) at 1 V potential for all the studied sample sets (**bottom right**).

A typical photoelectrochemical response of one of the printed electrodes under continuous UV irradiation and in the dark is depicted at Figure 8. The working electrode consisted of a 3-layered 1 cm^2^ circular patch printed on FTO glass substrate. Three complete cyclic scans were recorded in this measurement in order to confirm the stability and repeatability of the setup. The “light” curve describes the profile of the total photocurrent delivered by the device, *i.e*., the number of free charge carriers generated in the semiconductor and drawn into the external circuit by the applied voltage. The photocurrent steeply increases in the 0–0.5 V range, and then reaches a plateau where the current density is essentially independent on the voltage. If we compare samples of identical area and thickness, the photocurrent magnitude then becomes a comparative indicator of the semiconductor “quality”, *i.e*., its efficiency of charge carriers generation, separation and conduction. Since the samples were printed, their area and thickness is very well defined and reproducible and therefore we can use the measured photocurrent data to compare and evaluate various samples.

The figures depict that the photoresponse of printed green body cell is very poor, yielding approximately 8 µA/cm^2^. Such a low photocurrent can be attributed to a very high resistivity of the layer resulting from the limited contact between titania nanoparticles observed at the SEM images (Figure 5), which are surrounded by the insulating organosilica binder containing residual methyl groups [50]. Therefore further processing is necessary in order to induce mineralization of the layer and improving the semiconducting properties. Sintering ensures not only complete mineralization, but also titania crystallite redistribution and improved contact with the substrate conductor. The positive effect of sintering is obvious as the response curve reaches 160 µA/cm^2^ of photocurrent density. On the other hand, the UV-cured response curve delivered current density of 60 µA/cm^2^. While the UV-curing is capable of removing residual methyl groups by photocatalytic oxidation, no particle redistribution can be expected during this type of layer processing.

The overall performance of all samples is summarized and compared at Figure 8 where several trends can be easily identified: As one may expected, photocurrent density is increasing with titania content in all three studied processing options since the fraction of photosensitive component is increasing. Minor decrease of photocurrent density for Ti-5-sintered and Ti-5-UVcured samples can be attributed to reduced amount of ink ejected from the printing head due to blocked nozzle issues and/or layer wear during sample manipulation and washing caused by too low binder content and resulting compromised mechanical stability. For all titania-binder ratios, sintering resulted into approximately 2.5 times greater photocurrent values than in the case of UV-curing. So although sintering remains superior processing method if high photocurrents are desired, UV-curing provides a sound option for heat sensitive substrates.

### 2.9. Photocatalytic Activity

Terephtalic acid was used as a model aqueous pollutant for evaluation of photocatalityc activity of the printed samples. Hydroxyterephtalic acid is its main oxidation product which can be conveniently detected by its fluorescence peaking and 425 nm. During the course of this experiment, the fluorescence due to the intermediary product (OH-substituted) would first increase, slow down and eventually decrease down to zero as the fluorescent intermediate is further oxidized, following the typical kinetic profile of series reactions. The recorded fluorescence intensity is proportional to hydroxyterephtalic concentration and can be used directly as quantitative indicator if samples of the same size and thickness are compared.

**Figure 9:**
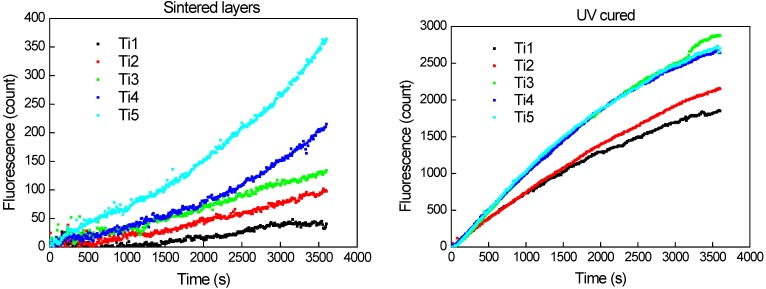
Records of hydroxyterephtalic acid fluorescence intensity *vs.* irradiation time for sintered (**left**) and UV-Cured (**right**) sample sets.

Samples used for this experiment consisted of 3-layered, 20 × 20 mm titania patch printed onto pyrex glass plate. Fluorescence intensity records plotted at Figure 9 show the initial phase of the reaction, *i.e*., the intermediate build-up. In the case of UV-cured sample set, the reaction proceeds faster and the slowing-down of the intermediate production rate becomes apparent during the experiment timeframe. UV-cured samples Ti_1 and Ti_2 exhibit lower initial rates than the others because of lower titania content in the layer. No difference in the reaction initial rate was observed for UV-cured samples Ti_3, Ti_4 and Ti_5, suggesting that the rate-controlling factor is other than titania content in the layer. On the other hand, the sintered samples exhibited much lower initial reaction rate. The influence of titania content on the initial reaction rate is more pronounced in this case as it consistently increases with increasing titania fraction.

The overall lower activity of sintered samples when compared to the UV-cured ones can be attributed to lower adsorption of aqueous reactant due to the hydrophobic nature of sintered samples demonstrated during the contact angle measurement. This suggestion is further supported by the shape of the fluorescence intensity curves recorded for the sintered samples: we actually see a gradual acceleration of the reaction, which is caused by *in-situ* UV-induced hydrophilization of the catalyst interface resulting into increasing adsorption of the aqueous substrate to be oxidized.

### 2.10. Print Quality Optimization

The above presented results indicate a promising potential of the studied material for a number of applications. Plain photocatalytic remediation of water has been convincingly demonstrated by the binder inventors [50] and confirmed by our experiment. However, the reported titania-silica composite represents a highly interesting material for photonic and electrophotocatalytic applications too, as demonstrated by our linear voltammetry experiments. Moreover, the UV-curing post-printing treatment option enables the deposition on organic flexible substrates and inkjet printing allows for direct patterning of arbitrary patterns. Such capability may be highly useful for the fabrication of electrochemical sensors [53,54], photochemical cells with special electrode geometry [55] or inverted structure polymer solar cells [56,57,58] because in all these devices patterned titania layers are needed.

In order to optimize the reported hybrid titania-silica coating for these advanced applications, further attempt to improve the quality of printed layers was made. The main goal was to improve the printed titania layer smoothness and remove banding artifacts. After analyzing the experimental results, we selected the formulation Ti_3, where the titania–silica volume ratio was 2.5:1.5, as the optimal printing mixture, since it delivered a balanced compromise of printing reliability, print quality, mechanical stability, electrical and photocatalytic properties.

A new batch of formulation Ti_3 was prepared in the same way as reported above, but milling with glass balls was prolonged to 3 days and the formulation was further diluted 1 + 1 by hexanol. The new ink was filled into the Dimatix cartridge and printed with the previously used settings. A line resolution test was printed onto FTO glass and the printouts were processed by UV-curing only, as this is the preferred processing option for flexible substrate based organic electronic structures. SEM imaging and profilometric analysis was performed in order to evaluate the resolution limit and surface quality of patterns printed in this way.

**Figure 10:**
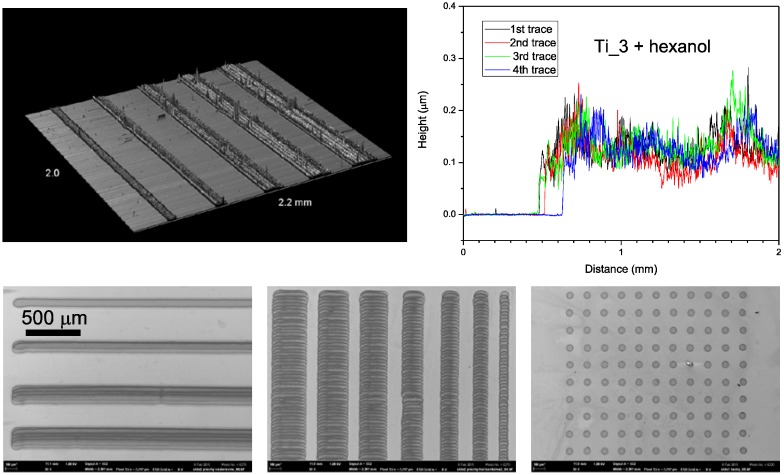
Profilometric mapping (**top**) and SEM images (**bottom**) of line resolution test printed with optimized Ti_3 formulation.

Figure 10 depicts the obtained results for several finest-line section of the resolution test. The prolonged milling and further ink dilution certainly contributed to the elimination larger aggregates which could get stuck in the nozzles and therefore no diverted droplets can be observed. The low volatility of hexanol enabled wet film leveling before drying, suppressed the banding issues and contributed to much smoother surface finish of the layers printed with the optimized composition. Although some protruding aggregates are still present and the individual droplet imprints are apparent, the print quality much improved both in terms of edge definition and smoother surface finish. With the optimized formulation, patterns with both positive and negative features down to 100 µm size are possible to print.

## 3. Experimental Section

### 3.1. Preparation of Silica Binder

The silica matrix was prepared through the sol-gel process at low temperature according the procedure described in work of Gregori *et al*. [50] where methyltriethoxysilane (MTEOS, ABCR) was used as the silica precursor. In the first step, MTEOS (60 cm^3^) was hydrolysed in acidic water (70 cm^3^) and the created mixture was continuously stirred for 1 h at laboratory temperature. Subsequently, the distillation at 115 °C was carried out to remove the released alcohol. The distillation was stopped in the moment of white suspension formation and immediately, 37 cm^3^ of cold water was added to increase the phase separation. After the cooling to laboratory temperature, diethyl ether (90 cm^3^) was added to the solution to extract the remaining water and subsequently the whole water phase was removed. Diethyl ether was evaporated under reduce pressure and finally, the rest matter was diluted in ethanol (50 cm^3^). The dry mass content was determined gravimetrically and adjusted to 0.28 g/cm^3^.

### 3.2. Printing Deposition

For the sake of convenient testing of various silica-titania ratios, stock dispersion of titanium dioxide in anhydrous ethanol was mixed first, having the same dry mass content as the silica binder solution. The printing formulation was then prepared by mixing varying volumes of silica binder solution, titania dispersion and viscosity-controlling solvent (butanol) in 20 mL glass vials. Approximately 3 g of 1 mm diameter glass balls were added to each vial and they were kept shaking for minimum 2 days on an oscillating plate shaker set to 900 rpm. Table 4 outlines the composition of all printing formulations.

**Table 4:** Composition of printing formulations.

Sample	Stock Dispersion of TiO_2_ in Ethanol (mL) c = 0.28 g/mL	Silica Sol in Ethanol (mL) c = 0.28 g/mL	Butanol (mL)	Titania Fraction in Dry Mass (wt %)
Ti_1	1.5	2.5	6	38
Ti_2	2	2	6	50
Ti_3	2.5	1.5	6	63
Ti_4	3	1	6	75
Ti_5	3.5	0.5	6	88

Printing of prepared inks was performed with an experimental inkjet printer Fujifilm Dimatix 2831 (Fujifilm Dimatix, Santa Clara, CA USA). The printer features a disposable 16-nozzle piezoelectric jetting printhead coupled with a 2 mL polyethylene ink tank. It is capable of printing on A4 size substrates with a resolution of up to 5080 dpi, *i.e*., 5 µm. Both substrate and printhead can be heated in order to speed up solvent evaporation and reduce ink viscosity, respectively. A stroboscopic camera provides still images or slow-down video for the observation of drop formation process, while another fiducial camera is used for precise substrate positioning and aligning of subsequently print layers. The printer has been successfully employed for the deposition of a wide variety of functional and auxiliary layers and during the past years has de facto become the industrial standard tool for ink development and testing.

Generally, the following procedure was repeated for each tested formulation: the prepared ink was loaded into a syringe and a blunt needle was attached to the syringe lure ports. The ink was filled into the Dimatix ink tank and the Dimatix 10 pL printing head was attached to the tank and the set was mounted into the Dimatix printer. The drop formation characteristics of all formulations were checked by means of the built-in stroboscopic camera and interaction of the printed material with the substrate was observed by an optical microscope. At the beginning of the preliminary testing period, the optimal printing conditions were found: Dimatix Model fluid 2 waveform, 18 V driving voltage, nozzle temperature 30 °C, substrate temperature 40 °C. Nozzle span was set to 30 μm (*i.e*., 33.3 drops per mm, 1111 drops per mm^2^). Printing was repeated up to 5 times to obtain different overall thicknesses of titania-silica layers. Complete drying took place after printing each layer, so that the following layer was printed in the “wet-to-dry” manner.

All tested ink formulations (see Table 4) were used for the fabrication of 5 parallel samples sets containing various test patches the size and shape of which were designed according to the experimental technique they were intended for. All the patterns in the set were printed in a range of thicknesses of 1 to 5 layers by repeated overprinting in a wet-to-dry manner, *i.e.* the previous layer was completely dry before printing the following one started. Actually, each sample set was printed in 3 copies to allow for various curing methods (see below). Simple rectangular patterns 20 × 20 mm were printed onto standard 26 × 76 mm Pyrex microscope slides for photocatalytic activity experiments. This pattern was also printed soda-lime microscope slides for hardness evaluation. A line resolution test pattern was printed onto the same substrate for profilometric and AFM investigation. A miniaturized version of the line resolution test was printed FTO coated glass slides for SEM imaging. 1 cm^2^ circular patch was printed onto FTO glass slide for photoelectrochemical measurements.

The deposition process was finalized by curing. One copy of each sample set let dry at ambient temperature and is further referred to as “printed” (P) sample set. The second one was calcined at 450 °C in air and is further referred to as “sintered” (S) sample set. The third one was immersed into demineralized water and placed 30 cm under an industrial processing UV lamp (model 80 BQL7, 248 W, Ultralight AG, Schaanwald, Liechtenstein) for 10 h and was called “UV cured” sample set (UV).

### 3.3. Characterization of Thin Films

The printed mixtures were examined using thermogravimetric analysis in order to understand the processes taking place during thermal processing. The analysis was performed in a thermoanalyser (TGA; 6300 TG-DTA, Seiko Instruments, Chiba, Japan) under the following parameters: a mixture of argon and air atmosphere (1:1 by volume), temperatures range of 35–1200 °C with temperature ramp of 5 °C/min up to 100 °C and afterwards 10 °C/min, and a flow rate of 400 cm^3^/min.

The hardness of the three layered samples was analyzed though the standard pencil hardness test [59]. Pencils with different hardness were successively placed into the pencil tester, from the hardest to the softest, and the hardness of pencil that will not cut into or gouge the film was evaluated as the hardness of the layer. The pencils were held in the tester firmly against the film at 45° angle and the speed of the tester was approximately 1 mm/s.

The thickness and roughness of the printed layers was evaluated using a DektakXT profilometer (Bruker, Billerica, MA, USA). In this analysis, a diamond tip got to the contact of sample surface and moved across the sample for the distance of 3 mm with a contact force of 5 mN. The vertical position of the diamond stylus generates an electric signal from which the final thickness is calculated. The roughness of the printed layers was calculated also from this measurement according the Formula (1) [60] where Rms means root mean square average, *z* are widths of peaks profile and *n* is number of peaks. The samples were scanned perpendicular to the direction of print head movement so that the printed bands merging could be evaluated:

(1) $$R_{RMS}=\sqrt{\frac{1}{n}\sum_{i=1}^{n}z^{2}}$$

The quality of prepared layers was investigated by an optical microscope Nikon Eclipse E200 connected with a D5000 digital camera (Nikon, Tokyo, Japan). The influence of silica amount in printing composition was examined and the obtained results served for evaluation of the best printing composition and conditions. The morphology of the samples was investigated by scanning electron microscopy (SEM, Ultra Plus, Carl Zeiss, Oberkochen, Germany) where the structure of different layers number as well as printed *vs.* sintered samples were compared. The topography of prepared samples was performed by atomic force microscopy (AFM, Dimension Icon, Bruker) in tapping mode. Each analysis was carried out in the center of the sample from area 1 μm^2^. Next the topology we investigated also roughness of the sample. In this case we found R_a_ which is arithmetic average of the absolute values defined by Equation (2) [61]:

(2) $$R_{a}=\frac{1}{l}\int_{0}^{l}\left|Z(x)\times dx\right|$$

The photoinduced hydrophilicity was studied through the contact angle measurement of water sessile droplet on the titania/silica surfaces by an OCA20 instrument (DataPhysics Instruments GmbH, Filderstadt, Germany). The total volume of deposited droplets was 5 μL and the records of droplets were taken in the 5 s after their formation. The wetting properties of printed, as well as sintered layers were examined. At first, the water droplet contact angle was measured on freshly prepared samples and afterwards each film was irradiated by UV light with intensity of 10 mW/cm^2^ (Lynx-L, Osram Sylvania, Danvers, MA, USA). The time required for conversion of properties from hydrophobic to hydrophilic was investigated. The final contact angle in certain time was calculated as the average of five measurements.

Photoelectrochemical characterization was performed by linear sweep voltammetry at room temperature using a two-electrode setup with the 1 cm^2^ titania patches. The printed FTO slide was scratched with a diamond knife and thus two isolated FTO strips were created. One strip with the printed titania patch served as the working electrode and the opposite naked FTO strip as the counter electrode. This setup was fitted into a custom build quartz cuvette. The cuvette was filled with 0.1 M phosphate buffer (pH = 7) and fitted onto an optical bench equipped with a fluorescent UV-A lamp emitting a broad peak centered at 365 (Sylvania Lynx-L 11 W). A magnetic stirrer was placed beneath the cuvette and a magnetic flea inside the cuvette provided efficient electrolyte mixing. The lamp emission was monitored by a X97 Irradiance Meter with a UV-3701 probe (Gigahertz Optic, Türkenfeld, Germany) and the irradiance was set to 3 mW/cm^2^ by adjusting the lamp-to-cuvette distance. Measurements of generated photocurrents were performed with an electrometer build on the basis of National Instruments Labview platform and supplying a linear voltage gradient of 10 mV/s from −0.5 to 2 V.

Photocatalytic experiments were conducted with the same cell and light source as the electrochemical response curves were measured with. Recently, terephtalic acid was suggested as a model compound for monitoring the oxidative activity of valence band holes generated in the immobilized photocatalyst [62]. Upon oxidation, presumably resulting from the attack of a hydroxyl radical, terephtalic acid is oxidized into hydroxyterephtalic acid, which gives a strong fluorescence signal at 425 nm. This approach proved to be very convenient for our experimental setup, because a single radiation source could be used for both the activation of the photocatalyst as well as the excitation of the fluorescent probe generated during the course of the reaction. The emitted fluorescence was collected by a quartz collimating lens mounted in the lateral wall of the cell holder and projected into an optical fiber attached to a Redtide spectrometer (Ocean Optics, Dunedin, FL, USA). The spectrometer driving software allowed for a convenient automated recording of the fluorescence intensity. Calibration was performed using hydroxyterepthalic acid standard (Sigma Aldrich, St. Louis, MO, USA).

## 4. Conclusions

The primary aim of this work was to find the optimal inkjet printable formulation and processing conditions for the fabrication of (electro)photocatalytic thin films consolidated by hybrid organosilicate binder. Several different ratios of titania and binder were tested and we were able to prepare semi-transparent homogeneous layers of arbitrary shape and thickness by repeated overprinting using all the studied compositions. The employed binder proved to have positive effect on titania particles stabilization and also acts as a lubricant enabling printing formulations with high solids loading (0.112 g/mL). Such concentrated inks can be effectively used for the fabrication of relatively thick coatings by a single pass of printing head which reduces printing time and eliminated artifacts originating from the interaction of wet and dry ink.

The printed green-body patterns exhibited good mechanical properties, but required further processing for a number of reasons. First, the hybrid organosilicate binder needed to be mineralized in order to become insoluble. Secondly, further processing is also necessary in order to improve the charge transfer properties and thirdly, it renders the catalyst surface superhydrophilic which is beneficial for photocatalytic activity. The post-printing processing is possible to carry out either by sintering at elevated temperature in oxidizing environment, or by UV curing through photocatalytic effect of the nanocrystalline titania.

All freshly printed films were superhydrophobic due to the residual methyl groups present in the binder. UV curing was found to induce both methyl groups mineralization as well as hydrophilic conversion. However, thermal sintering resulted only in methyl group mineralization, but sintered layers remained hydrophobic. Sintered layers can be converted to hydrophilic state by further UV curing, but their conversion towards hydrophilic properties takes more time than the conversion of green body printed films.

From the electrochemical point of view, the surface hydrophilicity does not seem to have much influence. Photocurrent density is increasing with titania content in all three studied processing options since the fraction of photosensitive component is increasing. For all tested formulations, sintering resulted into approximately 2.5 times greater photocurrent values than in the case of UV-cured electrodes. So while sintering remains the superior processing method for inorganic substrates, UV-curing provides a sound option for heat sensitive substrates.

As far as the photocatalytic activity is concerned, we discovered significant differences between the two studied post-printing processing options. Sintered layers showed very low activity, presumably due to poor adsorption of aqueous pollutants on their hydrophobic surface. However, acceleration of their activity was observed during the reaction, apparently as the surface was being converted into hydrophilic by the incident UV radiation during the course of the reaction. UV-cured sample series showed much higher activity and the kinetic profile of the first intermediate showed shape typical for the kinetic model of serial reactions.

Finally, sample Ti_3 in which the titania-silica volume ratio was 63 wt %, was evaluated as the best since it delivered a balanced compromise of mechanical stability, electrical and photocatalytic properties. We proceeded to our secondary experimental goal with this formulation, *i.e*., the optimization of print resolution and surface finish. The milling time was prolonged, the formulation was further diluted with hexanol and printing mode changed to single nozzle mode in order to provide maximum print quality. Electronic imaging and stylus profilometry confirmed that patterns with both positive and negative features down to 100 µm size are possible to print with this formulation and printing setup. The described technology is well suited for fabrication of various devices where thin patterned titania layers are needed.
